# Lopinavir to atazanavir or darunavir switch in HIV-1-infected patients with dyslipidemia: an observational study

**DOI:** 10.1186/1758-2652-13-S4-P46

**Published:** 2010-11-08

**Authors:** G Le Moal, G Beraud, T Prazuck, L Hocqueloux, A Dupuis, N Venisse

**Affiliations:** 1 CHU La Miletrie, COREVIH Centre-Poitou Charentes, Poitiers, France; 2 CHU La Miletrie, Poitiers, France; 3 CHR La Source, COREVIH Centre-Poitou Charentes, Orleans, France

## Background

Antiretroviral therapies including lopinavir (LPV) are frequently associated with dyslipidemia. Atazanavir (ATZ) and darunavir (DRV), currently recommended new boosted protease inhibitor, seems to have a better lipid profile than LPV, respectively in the Castle and Artemis study.

## Purpose of study

An observational study was conducted to evaluate the benefit of switching LPV to ATZ or DRV on the lipid results, and to compare the potency of DRV and ATZ to enhance the lipid profiles.

## Methods

Patients treated with LPV with undetectable viral load since 6 months were switched to ATZ or DRV if they have low density lipoprotein (LDL)>1.6 g/l with no other cardiovascular risk, LDL>1.3g/l with at least 1 cardiovascular risk, or high density lipoprotein (HDL)<0.35mmo/l. None of the patients had an additional lipid-lowering treatment since 3 months, and others antiretroviral treatment wasn't modified.

## Results

Forty two patients were included (12 females and 30 males), mean age 45.7 years (25-65). At baseline mean (SD) triglyceride (TG) level was 2.50g/l (1.64), total cholesterol (TC) 2.14g/l (0.51), LDL 1.23g/l (0.42) and HDL 0.48g/l (0.17). The two groups (26 ATZ and 16 DRV) were not different at baseline for weight, body mass index, CD4, TG, TC, LDL and HDL. At month 12, none of the 42 patients had virological failure but 1 patient in the DRV group had his treatment switched to Atripla® for simplification. There was a significant decrease in TG and TC, -0.54g/l (1.06) (p<0.01) and -0.15g/l (0.41)(p<0.05) respectively [mean (SD)]. In ATZ and DRV group, the variation was respectively -0.33g/l (1.11) and -0.91g/l (0.89) for TG (p=0.08), -0.17g/l (0.45) and -0.12g/l (0.36) for TC (p=0.75), -0.08g/l (0.37) and +0.04g/l (0.41) for LDL (p=0.47), +0.01g/l (0.11) and +0.03g/l (0.10) for HDL.

## Conclusion

In this observational study, switching LPV for ATZ or DRV enhance the lipid profile. DRV has a trend to reduce TG level more than ATZ.

